# A Case of Progressive Familial Intrahepatic Cholestasis Type–3

**DOI:** 10.7759/cureus.72782

**Published:** 2024-10-31

**Authors:** Jaladhi V Bhatt, Ambika Akhoury, Vaidehi V Vekaria, Hardik R Parmar, Dhruval N Patel

**Affiliations:** 1 Pediatrics and Neonatology, Narendra Modi Medical College and Sheth LG Hospital, Ahmedabad, IND; 2 Pediatrics, Shri Atal Bihari Vajpayee Medical College and Research Institute, Bangalore, IND; 3 Pediatrics and Neonatology, Carewell Trauma and Multispeciality Hospital, Kathlal, IND; 4 Pediatrics, Narendra Modi Medical College and Sheth LG Hospital, Ahmedabad, IND

**Keywords:** abcb4 gene, genetic mutation, hepatobiliary transporters, liver transplantation, progressive familial intrahepatic cholestasis (pfic)

## Abstract

Progressive familial intrahepatic cholestasis (PFIC) is a rare autosomal recessive disorder marked by severe, early-onset cholestasis due to genetic mutations in hepatobiliary transporters, leading to toxic bile acid accumulation and liver damage. PFIC is categorized into three types based on mutations in *ATP8B1*, *ABCB11*, and *ABCB4 *genes. This case involves a five-year-old female with symptoms such as easy fatigability, coarse facial features, respiratory distress, pruritus, abdominal distension, dark-colored urine, pale stool, and generalized edema. The patient, born to consanguineous parents with a family history of similar symptoms, showed severe pallor, icterus, clubbing, generalized edema, hepatomegaly, splenomegaly, and ascites on examination. Laboratory findings indicated severe anemia, thrombocytopenia, conjugated hyperbilirubinemia, low protein and albumin levels, and elevated liver enzymes. Imaging confirmed liver and spleen enlargement, ascites, and cardiomegaly. Genetic testing revealed a homozygous deletion in the* ABCB4* gene, diagnosing PFIC type 3. Treatment included ursodeoxycholic acid, fat-soluble vitamins, propranolol, intravenous albumin, fresh frozen plasma, and red cell transfusions. The patient was referred for further gastroenterological management and potential liver transplantation, and the family received genetic counseling. This case highlights the critical role of genetic testing in diagnosing and managing PFIC and the necessity of early identification and multidisciplinary care for such complex genetic disorders.

## Introduction

Progressive familial intrahepatic cholestasis (PFIC) is a rare group of autosomal recessive disorders characterized by early-onset, severe, and progressive cholestasis [1]. The prevalence of the disease varies from one in 50,000 to one in 100,000 [2]. The disorder is caused by genetic defects in specific hepatobiliary transporters, leading to the intracellular accumulation of toxic bile acids and subsequent liver cell injury. These genetic variations have been shown to play a significant role in the pathophysiology of various cholestatic syndromes, including acquired conditions such as intrahepatic cholestasis of pregnancy and drug-induced cholestasis.

Patients with PFIC often present with characteristic clinical features such as chronic cholestasis, distinctive facial features, vertebral malformations, growth and mental retardation, and hypogonadism [3]. Genetic testing is crucial for accurate diagnosis, as it can identify the specific gene defect responsible for the condition.

The underlying pathogenesis of progressive familial intrahepatic cholestasis is complex and involves the disruption of hepatobiliary transport systems, leading to the accumulation of bile acids within hepatocytes. Recent research has highlighted the importance of genetic factors in the susceptibility to developing cholestatic liver diseases, with mutations in canalicular transporter genes being strongly associated with progressive and benign forms of familial intrahepatic cholestasis [4].

Based on laboratory findings, liver histology, and causative genes, PFIC is classified into three types: PFIC 1, caused by mutations in the *ATP8B1* gene, encoding FIC 1 (Familial Intrahepatic Cholestasis 1) protein, PFIC 2, caused by mutations in the *ABCB11* gene (ATP-binding cassette family B) encoding the bile salt export pump (BSEP), and PFIC3, caused by mutations in the *ABCB4* gene encoding the Multidrug Resistance 3 (MDR3) protein on chromosome 7q21 [5]. Manifestations in PFIC 1 and PFIC 2 are apparent in the neonatal period, but PFIC 3 can present in infancy, childhood, or young adulthood [1,2]. Methods like those of next-generation sequencing and whole exome sequencing have allowed quick detection [6].

## Case presentation

Patient presentation and past history

A five-year-old female child, second in birth order, presented with easy fatigability, coarse facial features, respiratory distress, pruritus, abdominal distension, dark-colored urine, pale stool, and generalized edema. The patient was born out of a tertiary consanguineous marriage. There were no notable complications reported during the antenatal period or at birth. The patient also had a history of delayed developmental milestones. The patient's elder brother had a similar clinical history, suggesting a potential genetic component to the observed symptoms.

Clinical examination

**Figure 1:**
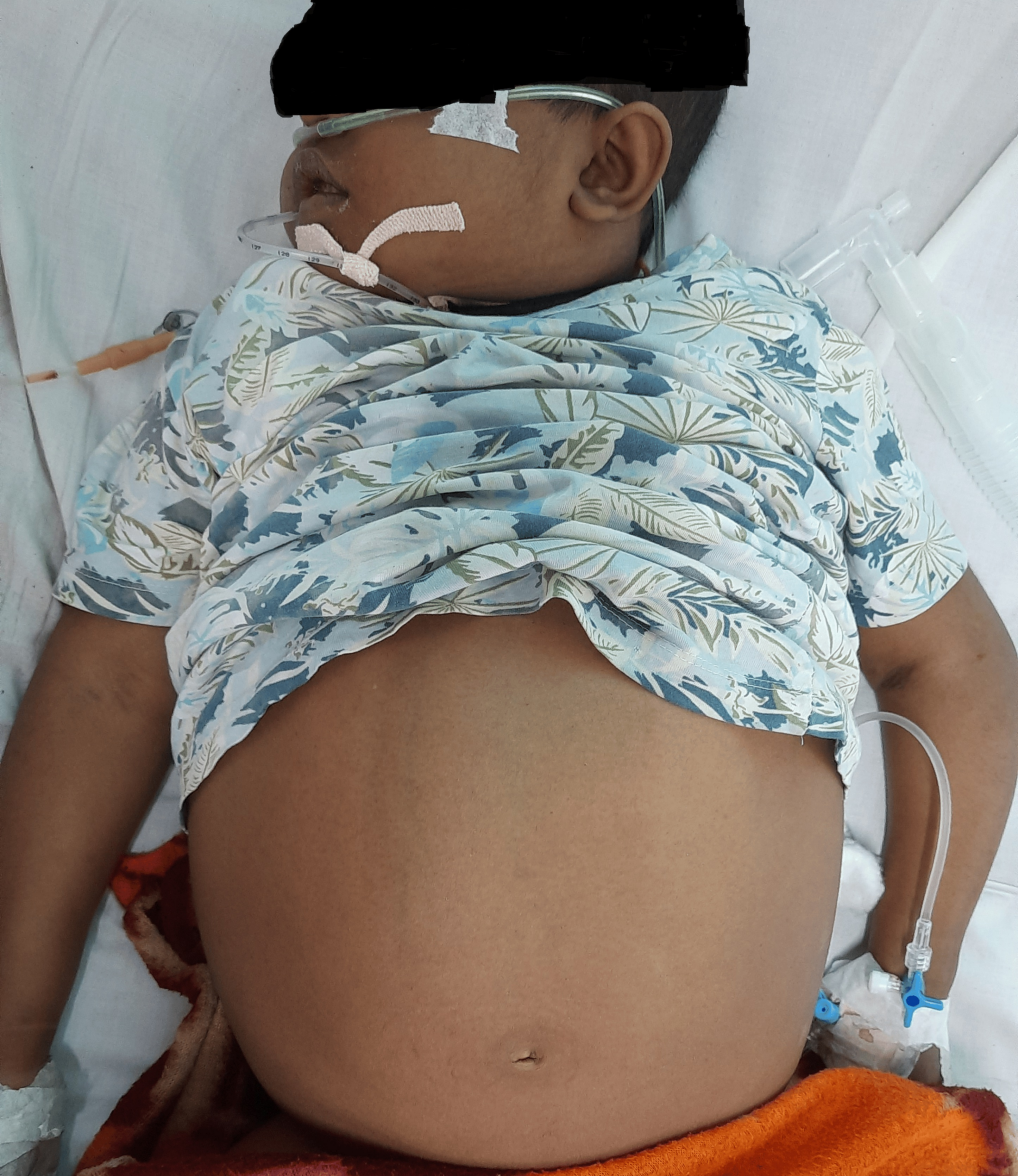
Five-year old female patient with edema and abdominal distension. Patient was put on oxygen with nasal prongs as a support for respiratory distress and Ryles tube was inserted to monitor altered gastric aspirate.

On anthropometric assessment, the patient's height was 104 cm, weight was 16 kg, BMI was 14.79 kg/m², and head circumference was 52 cm. General examination revealed severe pallor, icterus, clubbing, and generalized edema as shown in Figure 1. Vital signs included a heart rate of 120/minute, a respiratory rate of 46/minute (rapid and shallow), and blood pressure above the 90th percentile. Abdominal examination showed stretched, thin skin over the abdomen, a transverse and stretched umbilicus, visible veins, grade 2 splenomegaly and grade 3 hepatomegaly on palpation, and shifting dullness on percussion.

Laboratory and genetic investigations

All the abnormal laboratory investigations are shown in Table 1. Most of the investigations pertained to liver function tests; both synthetic and excreted products of the liver. Ultrasonography of the abdomen was done which showed altered liver echotexture with irregular nodular margins and increased periportal and pericholecystic echogenicity. Due to the typical symptoms and signs, imaging findings, and laboratory investigations directing to a liver pathology, the patient was advised for genetic investigation of exome sequencing. Table 2 shows the exome sequencing report which revealed the *ABCB4* gene abnormality.

**Table 1:** Laboratory Investigations SGPT: serum glutamate-pyruvate transaminase; ALT: alanine transaminase

Test Parameter	Patient Value	Typical Reference Range (Pediatric)
Hemoglobin	4 g/dL	11.5-15.5 g/dL
Platelet Count	1.34/μL	150,000-450,000/μL
Prothrombin Time (PT)	44.6 seconds	11-13.5 seconds
International Normalized Ratio (INR)	3.45	0.8-1.2
Total Bilirubin	1.4 mg/dL	0.3-1.2 mg/dL
Direct (Conjugated) Bilirubin	1.0 mg/dL	<0.3 mg/dL
Total Protein	5.2 g/dL	6.0-8.0 g/dL
Albumin	1.4 g/dL	3.5-5.5 g/dL
Cholesterol	71 mg/dL	120-200 mg/dL
SGPT (ALT)	224 IU/L	7-55 IU/L
Gamma Glutamyl Transferase (GGT)	296 IU/L	5-32 IU/L

**Table 2:** Exome Sequencing diagnosed the mutation in ABCB4 gene on Chromosome no. 7

Gene Transcript	Location	Variant	Zygosity	Disease	Inheritance	Classification
ABCB4(-)	Exons 16-23	c.(1893+1_1894-1)_(2924+1_2925-1)del (Exonic Deletion)	Homozygous	Progressive Familial Intraheptic Cholestasis-3	Autosomal Recessive	Likely Pathogenic

The medical management of this patient included a prescription of oral ursodeoxycholic acid (UDCA), fat-soluble vitamins, and propranolol. Supportive treatment included intravenous albumin, fresh frozen plasma, and packed red cell transfusions. She was referred to pediatric gastroenterology for further evaluation and management.

Parental guidance and follow-up

The relatives were counseled about the genetic nature of the disease and the potential need for liver transplantation as a definitive treatment. They were provided with detailed information regarding the ongoing care, including medication administration and the importance of regular follow-up visits to monitor the patient's condition and response to treatment. The family was also advised to seek genetic counseling to understand the implications of the condition for future offspring.

## Discussion

PFIC 3 is a rare autosomal recessive disease characterized by impaired bile flow due to mutations in the *ABCB4* gene, which encodes the MDR3 protein [2]. This condition affects both male and female patients equally and typically presents during infancy or early childhood [7]. PFIC 3 is a significant cause of liver disease in children, with approximately 41.1% of patients exhibiting an unfavorable prognosis [8].

In this case, the five-year-old female patient, born out of a consanguineous marriage (third degree), presented with a complex clinical picture indicative of PFIC 3. Her clinical manifestations included easy fatiguability, coarse facial features, respiratory distress, pruritis, abdominal distension, dark-colored urine, pale stool, and generalized edema. The presence of delayed developmental milestones and a similar clinical history in her elder brother, though not previously tested for any gene defect due to financial reasons, suggested a genetic etiology. In pathogenesis, PFIC 3 results from defects in canalicular phospholipase flippase, MRD 3 protein encoded by *ABCA4* gene, which results in deficient adenosine triphosphate (ATP)-dependent translocation of phosphatidylcholine across the canalicular membrane which inhibit neutralization of bile salt through micelle formation leading to damage to biliary epithelium and bile canaliculi. In PFIC 3, liver histology shows portal fibrosis, small bile duct hyperplasia, and mixed inflammatory infiltrates [9]. Children with PFIC 3 often present in the first year of life with clinical signs of cholestasis that lead to progressive liver disease and cirrhosis. Older children may have features of portal hypertension, including hematemesis and splenomegaly [10].

Genetic testing played a crucial role in confirming the diagnosis. Clinical exome sequencing revealed a homozygous contiguous deletion of exons 16-23 of the *ABCB4* gene at genomic location chr 7. The *ABCB4* gene encodes the MDR3 protein, a canalicular phospholipid flippase essential for the ATP-dependent translocation of phosphatidylcholine across the canalicular membrane. The mutation results in defective MDR3 protein function, leading to impaired phosphatidylcholine translocation. This impairment prevents the neutralization of bile salts through micelle formation, causing bile salt-induced damage to the biliary epithelium and bile canaliculi. Consequently, patients with PFIC 3 develop cholestasis, progressive liver disease, and cirrhosis. Histological findings typically include portal fibrosis, small bile duct hyperplasia, and mixed inflammatory infiltrates [11].

## Conclusions

This report details the case of a five-year-old female child with genetically diagnosed PFIC 3. Comprehensive genetic evaluation and targeted management strategies were implemented to address the immediate and long-term needs of the patient. The findings underscore the importance of early genetic testing and multidisciplinary care in managing rare disorders. Genetic counseling for the parent emphasized the significant risk of recurrence in future pregnancies, highlighting the necessity for ongoing monitoring and informed decision-making.
